# Effects of dexmedetomidine on renal microcirculation in ischemia/reperfusion-induced acute kidney injury in rats

**DOI:** 10.1038/s41598-021-81288-3

**Published:** 2021-01-21

**Authors:** Szu-Jen Yang, Chia-Ning Fan, Ming-Jiuh Wang, Shou-Zen Fan, Jui-Chang Tsai, Wei-Zen Sun, Wing-Sum Chan, Yu-Chang Yeh, NTUH Center of Microcirculation Medical Research (NCMMR), Ya-Jung Cheng, Yu-Chang Yeh, Chih-Min Liu, Po-Yuan Shih, Shih-Hong Chen, Ching-Tang Chiu, Anne Chao, Chun-Yu Wu

**Affiliations:** 1 Department of Anesthesiology, National Taiwan University Hospital, No. 7, Chung Shan S. Rd., Taipei City, 10002 Taiwan; 2 Institute of Medical Device and Imaging, College of Medicine, National Taiwan University, Taipei, Taiwan; 3 Department of Anesthesiology, Far Eastern Memorial Hospital, No. 21, Sec. 2, Nanya S. Rd., Banciao Dist., New Taipei, Taiwan

**Keywords:** Kidney, Kidney diseases, Acute kidney injury

## Abstract

Microcirculatory dysfunction plays a crucial role in renal ischemia/reperfusion (IR)-induced injury. Dexmedetomidine was reported to ameliorate IR-induced acute kidney injury. This study investigated the effects of dexmedetomidine on renal microcirculation after IR-induced acute kidney injury in rats. In total, 50 rats were randomly allocated to the following five groups (10 in each group): Sham, Control‒IR, Dex (dexmedetomidine) ‒Sham, Dex‒IR, and IR‒Dex group. The microcirculation parameters included total small vessel density, perfused small vessel density (PSVD), proportion of perfused small vessels, microvascular flow index, and tissue oxygen saturation (StO_2_) were recorded. The repeated measures analysis showed that PSVD on renal surface was higher in the Dex‒IR group than in the Control‒IR group (3.5 mm/mm^2^, 95% confidence interval [CI] 0.6 to 6.4 mm/mm^2^, *P* = 0.01). At 240 min, StO_2_ on renal surface was lower in the Control‒IR group than in the Sham group (– 7%, 95% CI − 13 to − 1%, *P* = 0.021), but StO_2_ did not differ significantly among the Sham, Dex‒IR, and IR‒Dex groups. Our results showed that pretreatment with dexmedetomidine improved renal microcirculation in rats with IR-induced acute kidney injury. However, the adverse effects of low mean arterial pressure and heart rate might offset the protective effect of dexmedetomidine on organ injury.

## Introduction

Acute kidney injury that resulted from shock and ischemia/reperfusion (IR)-induced injury is a major challenge in critical care medicine. Microcirculatory dysfunction plays a crucial role in IR-induced injury^1,2^. Many treatments were investigated to improve microcirculation^3–5^. Dexmedetomidine is a potent selective alpha-2-adrenergic agonist with sedative and analgesic effects^6,7^. Dexmedetomidine was proven to improve microcirculation in a septic rat model^8^, in a surgical stress and pain rat model^9^, and in patients after cardiac surgery^10^. In addition, dexmedetomidine ameliorates renal IR-induced injury^11^, reduces oxidative stress, and decreases kidney-injury-related biomarker levels^11–13^; however, some of these treatment effects remain controversial. To our knowledge, few studies were conducted on animals to investigate the effects of dexmedetomidine in terms of renal microcirculation on rats with renal IR-induced injury. This study investigated the effects of dexmedetomidine in terms of renal microcirculation on rats with renal IR-induced injury. Our primary outcome was the investigation of renal microcirculation at 240 min. For secondary outcome measurement, at 240 min, a blood test and enzyme-linked immunosorbent assay (ELISA) were performed to investigate biochemical laboratory data and kidney-injury-related molecules.

## Results

### Hemodynamic parameters

**Figure 1:**
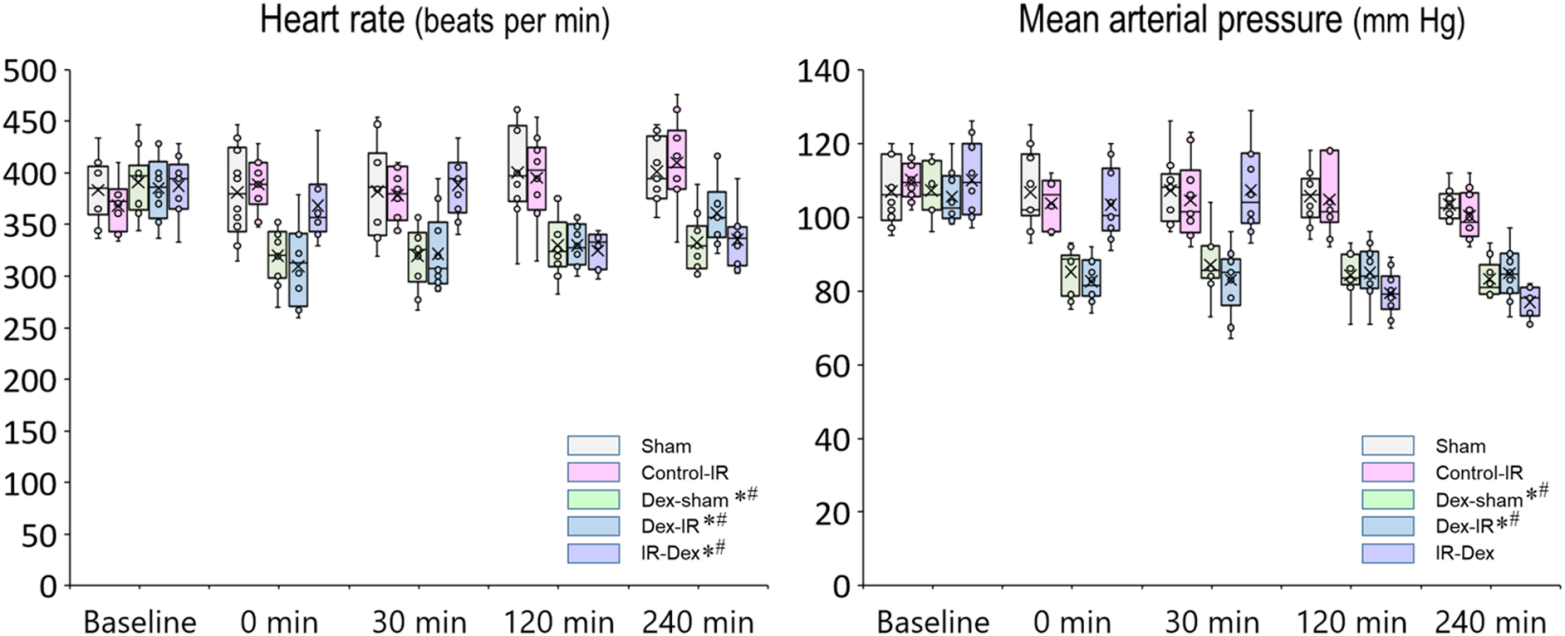
Mean arterial pressure and heart rate. *P < 0.05 vs. the Sham group determined using repeated measures analysis. ^#^*P* < 0.05 vs. the Control‒IR group determined using repeated measures analysis. X indicates the mean of each group. Sham indicates sham operation; Control‒IR (ischemia/reperfusion), left kidney ischemia for 60 min and reperfusion; Dex (dexmedetomidine)‒Sham, sham operation and infusion of dexmedetomidine; Dex‒IR, pre-ischemia infusion of dexmedetomidine 30 min before left kidney ischemia; IR‒Dex, post-ischemia infusion of dexmedetomidine 30 min after reperfusion of the left ischemic kidney.

Repeated measures analysis showed that mean arterial pressure (MAP) was lower in the Dex‒Sham, Dex‒IR, and IR‒Dex groups than in the Sham (– 17 mm Hg, 95% confidence interval [CI] − 24 to − 9 mm Hg, *P* < 0.001; – 18 mm Hg, 95% CI  − 25 to − 10 mm Hg, *P* < 0.001; – 11 mm Hg, 95% CI  − 18 to − 3 mm Hg, *P* = 0.001; respectively; Fig. 1A) and Control‒IR groups (– 15 mm Hg, 95% CI  − 23 to − 8 mm Hg, *P* < 0.001; – 16 mm Hg, 95% CI  − 24 to − 9 mm Hg,  *P* < 0.001; – 9 mm Hg, 95% CI  − 16 to − 2 mm Hg, *P* = 0.008; respectively; Fig. 1A). Repeated measures analysis showed that heart rates were lower in the Dex‒Sham and Dex‒IR groups than in the Sham (– 51 beats per minute [bpm], 95% CI  − 86 to − 16 bpm, *P* = 0.001; – 46 bpm, 95% CI − 81 to − 12 bpm, *P* = 0.004; Fig. 1B) and Control‒IR groups (– 50 bpm, 95% CI  − 85 to − 15 bpm, *P* = 0.002; – 45 bpm, 95% CI − 80 to − 10 bpm, *P* = 0.006; respectively; Fig. 1B).

### Microcirculatory dysfunction and lower tissue oxygen saturation (StO_2_) after ischemia/reperfusion injury

**Figure 2:**
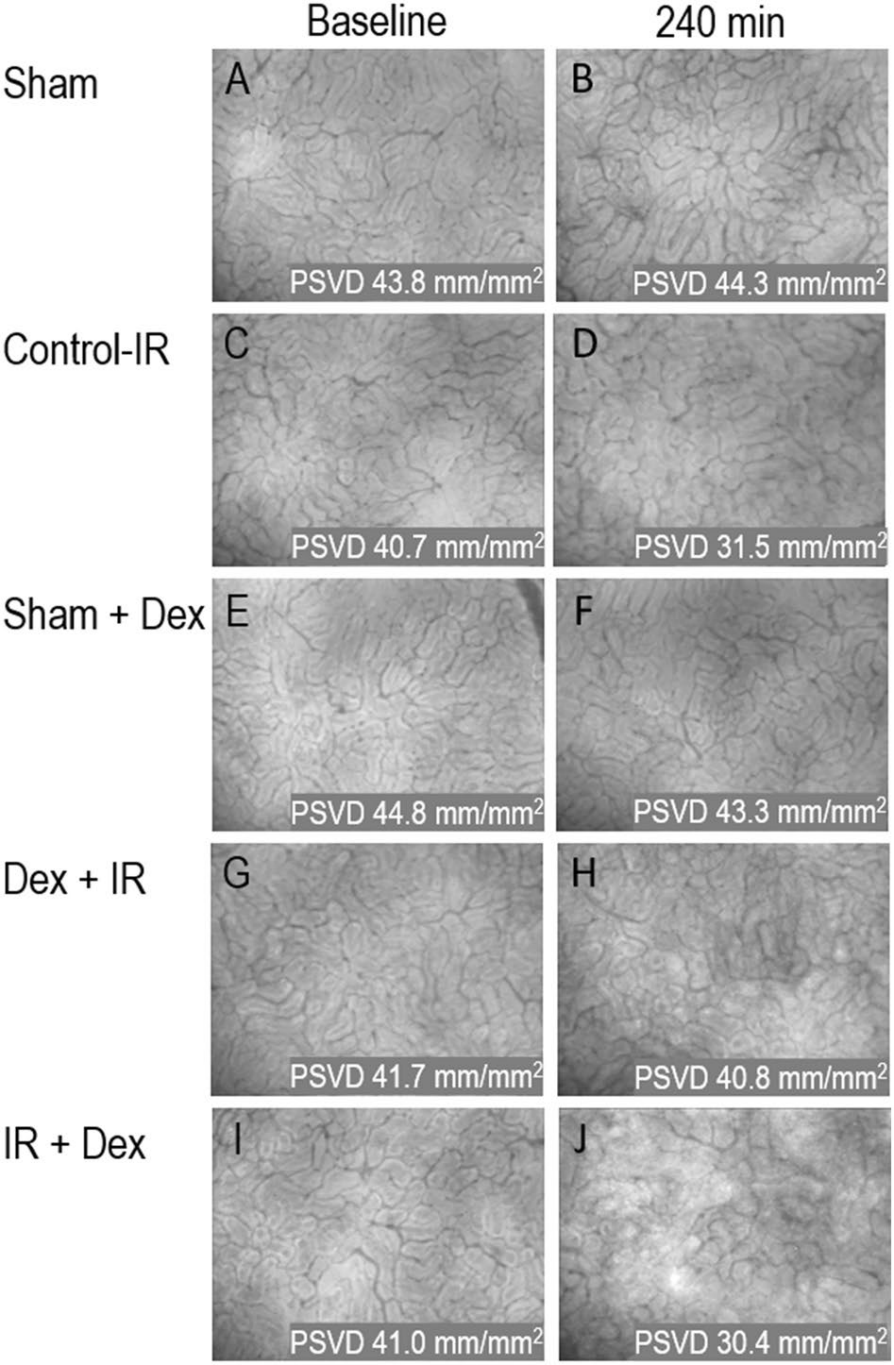
Microcirculation images of the renal surface from a sidestream dark field video microscope at baseline and 240 min after ischemic kidney reperfusion. (**A**, **C**, **E**, **G**, **I**) baseline of the five groups. (**B**, **D**, **F**, **H**, **J**) At 240 min after ischemic kidney reperfusion. At 240 min, perfused small vessel density was lower in the Control‒IR and IR‒Dex groups than in the Sham group. Sham indicates sham operation; Control‒IR (ischemia/reperfusion), left kidney ischemia for 60 min and reperfusion; Dex (dexmedetomidine)‒Sham, sham operation and infusion of dexmedetomidine; Dex‒IR, pre-ischemia infusion of dexmedetomidine 30 min before left kidney ischemia; IR‒Dex, post-ischemia infusion of dexmedetomidine 30 min after reperfusion of the left ischemic kidney. PSVD, perfused small vessel density.

**Figure 3:**
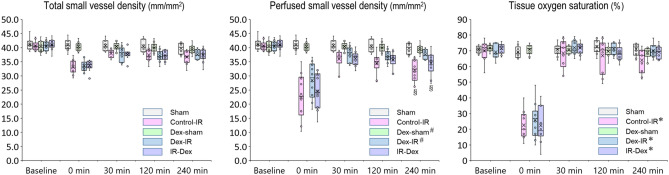
Total small vessel density, perfused small vessel density, and tissue oxygenation saturation. *P < 0.05 vs. the Sham group and ^#^*P* < 0.05 vs. the Control‒IR group determined using repeated measures analysis. §*P* < 0.05 vs. the Sham group at 240 min determined using one-way analysis of variance with Tukey’s test. X indicates the mean of each group. Sham indicates sham operation; Control‒IR (ischemia/reperfusion), left kidney ischemia for 60 min and reperfusion; Dex (dexmedetomidine)‒Sham, sham operation and infusion of dexmedetomidine; Dex‒IR, pre-ischemia infusion of dexmedetomidine 30 min before left kidney ischemia; IR‒Dex, post-ischemia infusion of dexmedetomidine 30 min after reperfusion of the left ischemic kidney.

The microcirculation images of the renal surface from the SDF video microscope at baseline and 240 min after ischemic kidney reperfusion are shown in Fig. 2. In Fig. 3, repeated measures analysis show that perfused small vessel density (PSVD) on renal surface was lower in in the Control-IR group than the Sham group (– 7.7 mm/mm^2^, 95% CI  − 10.7 to  − 5.0 mm/mm^2^, *P* < 0.001). In Fig. 3C, repeated measures analysis indicated that tissue oxygen saturation (StO_2_) on renal surface was lower in the Control‒IR group than in the Sham group (– 12%, 95% CI  − 17 to − 8%, *P* < 0.001). At 240 min, StO2 was lower in the Control‒IR group than in the Sham group (– 7%, 95% CI  − 13 to − 1%, *P* = 0.021). PSVD and StO_2_ on renal surface did not differ significantly between the Sham and Dex‒Sham groups.

### Treatment effect of dexmedetomidine on ischemia/reperfusion microcirculatory dysfunction

Repeated measures analysis for our primary outcome indicated that PSVD was higher in the Dex‒IR group than in the Control‒IR group (3.5 mm/mm^2^, 95% CI 0.6 to 6.4 mm/mm^2^, *P* = 0.01; Fig. 3B), but PSVD did not differ significantly between the IR‒Dex and Control‒IR groups (1.5 mm/mm^2^, 95% CI − 1.3 to 4.4 mm/mm^2^, *P* = 0.564; Fig. 3B). At 240 min, PSVD on renal surface was higher in the Dex‒IR group than in the Control‒IR group (6.5 mm/mm^2^, 95% CI 1.9 to 11.0 mm/mm^2^, *P* = 0.002), but PSVD on renal surface did not differ significantly between the IR‒Dex and Control‒IR groups (3.4 mm/mm^2^, 95% CI − 1.2 to 8.0 mm/mm^2^, *P* = 0.237). At 240 min, microvascular flow index (MFI), and proportion of perfused small vessels (PPSV) on renal surface were higher in the Dex‒IR group than in the Control‒IR group [3.0 (3.0–3.0) vs. 2.6 (2.3–2.9), *P* < 0.001; 100 (100–100) vs 89 (78–94)%, *P* < 0.001; respectively], but MFI and PPSV did not differ significantly between the IR‒Dex and Control‒IR groups [2.7 (2.5–2.9) vs. 2.6 (2.3–2.9), *P* = 0.353; 93 (88–98) vs. 89 (78–94)%, *P* = 0.190; respectively]. The microcirculation parameters of terminal ileal mucosa did not differ significantly among the five groups.

### Treatment effect of dexmedetomidine on StO_2_ after ischemia/reperfusion injury

In Fig. 3C, repeated measures analysis indicated that StO_2_ on renal surface did not differ significantly among the Control‒IR, Dex‒IR, and IR‒Dex groups, and StO_2_ was lower in the Dex‒IR and IR‒Dex groups than in the Sham group (− 9%, 95% CI − 13 to − 5%, *P* < 0.001; − 10%, 95% CI − 14 to − 6%, *P* < 0.001; respectively). At 240 min, StO_2_ on renal surface did not differ significantly among the Sham, Dex‒IR, and IR‒Dex groups. StO_2_ of terminal ileal mucosa did not differ significantly among the five groups.

### Histologic score and laboratory data

**Figure 4:**
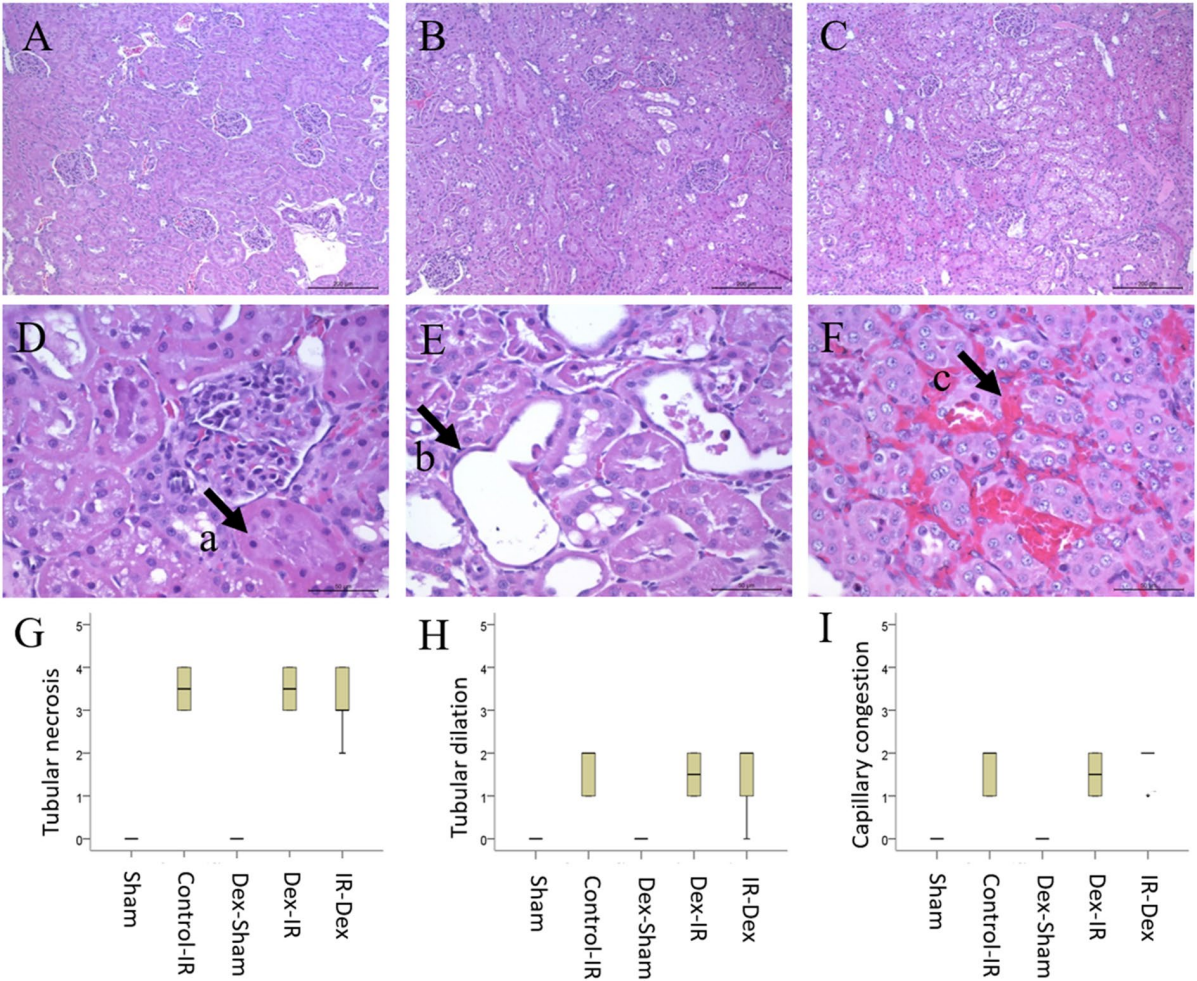
Histologic changes of ischemic/reperfusion injury in kidney. (**A**) Sham group; (**B**) Control-IR group; (**C**) Dex-IR group; (**D**) arrow a represents renal tubular necrosis; (**E**) arrow b represents renal tubular dilation; (**F**), arrow c, represents peritubular capillary congestion in outer medulla; (**G**), histologic scores of renal tubular necrosis; (**H**) histologic score of renal tubular dilation; (**I**) histologic scores of peritubular capillary congestion in outer medulla, * Two rats scores 1 in the IR-Dex group. Sham indicates sham operation; Control‒IR (ischemia/reperfusion), left kidney ischemia for 60 min and reperfusion; Dex (dexmedetomidine)‒Sham, sham operation and infusion of dexmedetomidine; Dex‒IR, pre-ischemia infusion of dexmedetomidine 30 min before left kidney ischemia; IR‒Dex, post-ischemia infusion of dexmedetomidine 30 min after reperfusion of the left ischemic kidney.

In Fig. 4, the histologic scores of renal tubular necrosis, renal tubular dilation, and peritubular capillary congestion in outer medulla did not differ significantly among the Control‒IR, Dex‒IR, and IR‒Dex groups. The blood test results for biochemical laboratory data are presented in Table 1. Although aspartate aminotransferase (AST) was statistically significant by an one-way ANOVA, further post hoc analysis showed that AST level did not differ significantly among the Dex‒IR, IR‒Dex, and Control‒IR groups. No significant difference was observed in creatinine, alanine aminotransferase (ALT), neutrophil gelatinase-associated lipocalin (NGAL), monocyte chemoattractant protein-1 (MCP-1), and kidney injury molecule-1 (KIM-1) levels among the five groups (Table 1).

**Table 1:** Laboratory and enzyme-linked immunosorbent assay results obtained at 240 min.

	Sham	Control-IR	Dex-Sham	Dex-IR	IR-Dex	*P* value
AST (U/L)	73 (11)	159 (32)	77 (11)	182 (34)	152 (26)	< 0.01
ALT (U/L)	26 (4)	30 (8)	24 (5)	27 (3)	27 (8)	0.45
Creatinine (mg/dL)	0.1 (0.1–0.1)	0.4 (0.3–0.4)	0.3 (0.2–0.3)	0.4 (0.4–0.5)	0.4 (0.4–0.4)	0.06
NGAL (ng/mL)	2077 (1129)	3216 (1442)	2047 (1281)	3132 (1867)	2043 (1319)	0.13
MCP-1 (pg/mL)	508 (166)	668 (359)	802 (412)	999 (933)	634 (400)	0.29
KIM (pg/mL)	825 (163)	886 (191)	871 (140)	954 (256)	1007 (328)	0.41
Lactate (mmol/L)	0.9 (0.2)	1.0 (0.3)	1.0 (0.3)	0.9 (0.2)	0.8 (0.2)	0.28

Data are presented as mean (SD), except for creatinine which is presented as median (interquartile range); n = 10 for each group. AST was found to be statistically significant based on an one-way analysis of variance, but further post hoc analysis using Tukey’s test showed that AST did not differ significantly among the Dex‒Sham, Dex‒IR, IR‒Dex and Control‒IR groups. Sham indicates sham operation; Control‒IR, left kidney ischemia for 60 min and reperfusion; Dex‒Sham, sham operation and infusion of dexmedetomidine; Dex‒IR, pre-ischemia infusion of dexmedetomidine 30 min before left kidney ischemia; IR‒Dex, post-ischemia infusion of dexmedetomidine 30 min after reperfusion of the left ischemic kidney; AST, aspartate aminotransferase; ALT, alanine aminotransferase; MCP-1, monocyte chemoattractant protein-1; NGAL, neutrophil gelatinase-associated lipocalin; KIM-1, kidney injury molecule-1.

## Discussion

Our result indicated that pretreatment with dexmedetomidine preserved renal microcirculation after IR-induced acute kidney injury, whereas post-treatment with dexmedetomidine did not. However, neither pretreatment nor post-treatment significantly decreased the injury biomarker levels. Additionally, dexmedetomidine lowered the MAP and heart rate of rats in this study.

Several possible mechanisms might explain why dexmedetomidine could preserve renal microcirculation. First, dexmedetomidine is an alpha-2 agonist, which can cause vasodilation of small vessels, reduce sympathetic activity, and maintain renal microvascular blood flow^14^. This protective effect of dexmedetomidine on renal microcirculation in rabbits with renal IR injury was reported in the study of Si et al.^15^, dexmedetomidine can increase the tissue level of superoxide dismutase and increase the antioxidant effect against the oxidative stress resulting from IR injury^13,16,17^. Moreover, dexmedetomidine has an anti-inflammatory effect through alpha-2 adrenergic receptors, which can reduce the level of inflammatory mediators resulting from IR-induced injury^18,19^. Antioxidant and anti-inflammatory effects of dexmedetomidine protect the endothelium of small vessels and attenuate the severity of microcirculatory dysfunction. Third, dexmedetomidine has anticoagulation properties, which can prevent microthrombosis of small vessels and thus maintain microvascular blood flow^20^.

Whether dexmedetomidine can decrease kidney-injury-related biomarkers remains debatable. Several studies revealed that dexmedetomidine decreases kidney-injury-related biomarkers such as NGAL^21^, KIM-1^22^, and MCP-1^23^. Conversely, Bayram et al.^24^ and de Carvalho et al.^12^ found that dexmedetomidine did not reduce NGAL levels. Moreover, our study did not identify a significant decrease in NGAL, KIM-1 and MCP-1 levels. The lower dose of dexmedetomidine administered (3 mcg/kg/h) in our study is a possible reason. Other postulations relate to different doses of dexmedetomidine, different methods of dexmedetomidine administration, and the use of unilateral or bilateral renal artery clamping. For example, Lempiainen et al. proved that preconditioning bolus with a high dexmedetomidine dose (10 mcg/kg) ameliorates renal IR-induced injury^11^. In the study of Si et al., a high dose of dexmedetomidine (25 mcg/kg) was aministered through intraperitoneal injection^23^.

Bradycardia and hypotension are the most common side effects of dexmedetomidine^7^. Low MAP and slow heart rate in the three groups with dexmedetomidine treatment may cause some detrimental effects to the rat kidneys. First, dexmedetomidine might blunt the compensated tachycardia as a result of shock. In our previous endotoxemic rat model, the anti-sympathetic effect of dexmedetomidine reduced excessive tachycardia. In addition, dexmedetomidine can preserve intestinal microvascular blood flow and reduce intestine injury^8^. However, in this study, we found that although renal microvascular blood flow was preserved, the kidney injuries were not reduced. Thus, the kidney may require high microvascular blood flow and heterogeneity was observed in the blood flow among different organs. Second, renal perfusion is highly pressure dependent^25^, a dexmedetomidine‒related low MAP level may result in inadequate perfusion pressure while microvascular blood flow is preserved. Inadequate perfusion pressure may cause further kidney injury. It is suggested that researchers of further studies consider combining the administration of dexmedetomidine with vasopressor infusion or fluid supplement to maintain adequate perfusion pressure.

This study has several limitations. First, the sample size was too small to determine the differences of secondary outcomes among the five groups. Each secondary outcome may require different sample sizes to obtain adequate power. Second, observation of the renal surface microcirculation was limited by the instrument used. Advanced instruments or contrast-enhanced ultrasound may help us investigate microcirculation in the deeper kidney layers. Third, the severity of distant organ damage after renal IR-induced injury in this study was mild. Increasing the clamping time of the left renal artery or clamping of the bilateral renal arteries might be considered for investigating severer distant organ damage after renal IR-induced injury. Fourth, only male rats were used in this study. Gender difference of the effect of dexmedetomidine was reported between male and female patients^26^. Further studies are required to investigate the influence of gender on the microcirculation protective effect of dexmedetomidine. Fifth, circulatory levels of KIM-1, MCP-1, and NGAL did not differ significantly after treatment with dexmedetomidine. Immunohistochemistry for these markers were not available in this study, and further studies are required to investigate whether the tissue levels of these injury biomarkers differ significantly after treatment with dexmedetomidine.

In conclusion, this study demonstrates that pretreatment with dexmedetomidine before IR-induced injury could preserve renal surface microcirculation. However, the adverse effects of hypotension and bradycardia might offset the protective effect of dexmedetomidine on organ injury. Further studies of investigating the protective effects of dexmedetomidine are suggested to consider treating the adverse effects of hypotension and bradycardia.

## Methods

### Rats and ethics statement

In total, 50 male Wistar rats (body weight 250 ± 50 g; Biolasco Taiwan, Taipei, Taiwan) were used in this study. This study was approved by the Institute Animal Care and Use Committee of National Taiwan University (No. 20170071, College of Medicine, National Taiwan University, Taipei, Taiwan). The rats were handled according to the Ethical Guidelines for the Treatment of Laboratory Animals of the Institute Animal Care and Use Committee of National Taiwan University and kept on a 12-h light/dark cycle and had free access to water and food.

### Grouping

**Figure 5:**
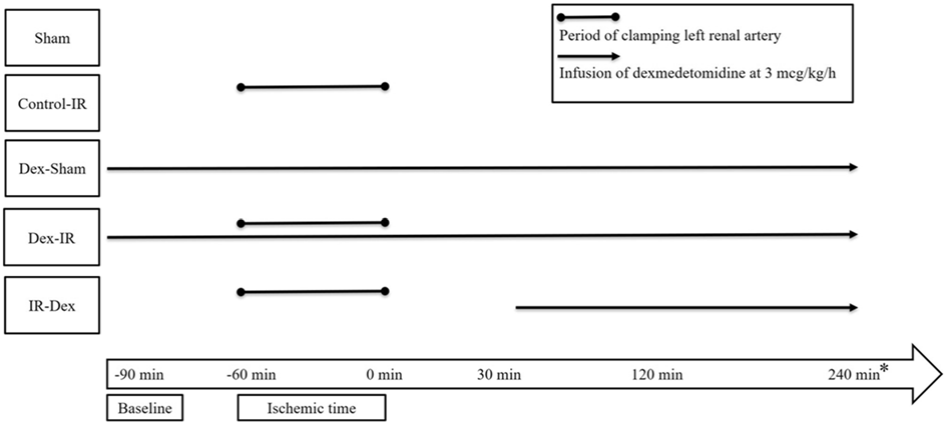
Time course of interventions for different groups. Ten rats were allocated to each group. Microcirculation were examined at baseline and 0, 30, 120, and 240 min. *Time point of the blood test. Sham indicates sham operation; Control‒IR (ischemia/reperfusion), left kidney ischemia for 60 min and reperfusion; Dex (dexmedetomidine)‒Sham, sham operation and infusion of dexmedetomidine; Dex‒IR, pre-ischemia infusion of dexmedetomidine 30 min before left kidney ischemia; IR‒Dex, post-ischemia infusion of dexmedetomidine 30 min after reperfusion of the left ischemic kidney.

These 50 rats were randomly allocated to the following five groups (with 10 rats in each group) (Fig. 5.): (1) *Sham*: sham operation, (2) *Control‒IR*: left kidney ischemia for 60 min and reperfusion, (3) *Dex (dexmedetomidine)‒Sham*: sham operation and infusion of dexmedetomidine (3 mcg/kg/h), (4) *Dex‒IR*: pre-ischemia infusion of dexmedetomidine (3 mcg/kg/h) 30 min before left kidney ischemia, and (5) *IR‒Dex*: post‒ischemia infusion of dexmedetomidine 30 min after reperfusion of the left ischemic kidney.

### Anesthesia, surgical procedure, and ischemic kidney model

The anesthetic procedure was similar to that of our previous related study^9^. Anesthesia was induced with 4% isoflurane by using an induction chamber connected to an animal anesthesia machine (Midmark, Orchard Park, NY, USA). After a rat was anaesthetized, it was placed in a supine position on an animal warming pad. Then, isoflurane was adjusted to 2% to maintain the anesthetic state. Intramuscular injection of 0.05 mg/kg atropine sulfate was administered to reduce respiratory tract secretion and block vagal reflexes elicited through intestinal viscera manipulation. Tracheostomy was performed, and a 14-G catheter (Surflo; Terumo Corporation, Laguna, Philippines) was inserted into the trachea. Subsequent anesthesia was maintained using 1.0% isoflurane. Polyethylene catheters (PE-50; Intramedic 7411, Clay Adams, Parsippany, NJ, USA) were inserted into the right common carotid artery and external jugular vein. The right common carotid artery catheter was used to continuously monitor arterial blood pressure and heart rate, and the external jugular vein catheter was used for 0.9% saline infusion at a rate of 2 mL/kg/h. Body temperature was continuously monitored. A 3-cm-long midline laparotomy was performed to exteriorize the left kidney for baseline microcirculation examination with a sidestream dark-field (SDF) video microscope and a tissue oxygen monitor (moorVMS‒OXY). Thirty minutes after baseline microcirculation was examined, the left renal artery was clamped for 60 min in the Control‒IR, Dex‒IR, and IR‒Dex groups. The arterial clamp was removed after 60 min, and left kidney reperfusion was observed for 240 min. MAP, heart rate, and subsequent microcirculation examinations were performed at 0, 30, 120, and 240 min after arterial clamp removal. At 240 min, a 2-cm-long sectioning was performed on the antimesenteric aspect of a segment of the terminal ileum, 6‒10 cm from the ileocecal valve, and the exposed mucosa was used for microcirculation examination. After the final examination, blood samples were collected for further laboratory and ELISA tests. Both kidneys were extracted for histologic examinations. Afterwards, the rats was euthanized through exsanguination cardiac arrest under anesthesia.

### Protocol of dexmedetomidine treatment

In the Dex‒Sham and Dex‒IR groups, dexmedetomidine was infused at a rate of 3 mcg/kg/h after baseline microcirculation examination, and the infusion fluid rate was controlled at 2 mL/kg/h. The inspired concentration of isoflurane was adjusted from 1.0% to 0.7%. In the IR‒Dex group, dexmedetomidine was infused at rate of 3 mcg/kg/h 30 min after the arterial clamp was removed, and the infusion fluid rate was controlled at 2 mL/kg/h. The inspired concentration of isoflurane was adjusted from 1.0% to 0.7%.

### Measurement of microcirculation and tissue oxygen saturation

SDF video microscope (MicroScan, Microvision Medical, Amsterdam, The Netherlands) was used to record microcirculation images of the kidney surface. At each time point, three continuous image sequences (10 s) were digitally stored for each measured site, and data for the three images were averaged for statistical calculations. The images were analyzed using semi-automated analysis software (AVA 3.0, Academic Medical Center, University of Amsterdam, Amsterdam, the Netherlands)^27^. TSVD, PSVD, PPSV, and MFI score were obtained according to suggestion from a microcirculation round table conference^27^. The analyses were conducted by a single investigator who was blinded to grouping. StO_2_ of the left kidney surface was measured using white light reflectance spectroscopy (tissue oxygen monitor, moorVMS‒OXY, Moor Instruments Ltd, United Kingdom) at the same time points(baseline, and 0, 30, 120, and 240 min). At each time point, three random sites of the measured area on the left kidney surface were examined, and data for these sites were averaged for statistical calculations. StO_2_ of the terminal ileal mucosa was measured 240 min after the arterial clamp was removed.

### Laboratory test and histologic scores

The blood samples obtained at 240 min were used to measure the serum level of ALT, AST, lactate, and creatinine. NGAL, MCP-1, and KIM-1 assays were performed using ELISA kits according to manufacturers’ instructions. The kidneys were sent to the Laboratory Animal Center, College of Medicine, National Taiwan University, for histologic analysis. In the pathology report, the severity of renal tubular necrosis, renal tubular dilation, and congestion of peritubular capillary in outer medulla were defined using histologic scores as follows: 1 = minimal (< 1%); 2 = slight (1‒25%); 3 = moderate (26‒50%); 4 = moderate/severe (51‒75%); and 5 = severe/high (76‒100%)^28^.

### Statistical analysis

Data analysis was conducted using statistical software (SPSS 20; IBM SPSS, USA). Continuous variables with a normal distribution were presented as mean ± standard deviation. Differences in means over time among the five groups were analyzed through repeated measures analysis followed by a post hoc analysis using Tukey’s test. Furthermore, differences in means at 240 min among the five groups were analyzed through an one-way analysis of variance (ANOVA) followed by a post hoc analysis using Tukey’s test. The primary outcome of this study was to compare PSVD among the five groups. This study was powered (10 rats for each group) to detect a 9% difference in PSVD on kidney surface at 240 min after reperfusion of ischemic kidney, with a α level of 0.05 and a β level of 0.2, assuming a control PSVD of 40 ± 2.2 mm/mm^2^ based on the result of our pilot study. A *P* value of < 0.05 was considered to indicate statistical significance. Non-normally distributed data, including creatinine and histologic scores were presented as median (interquartile range) values among the five groups and analyzed using the Kruskal‒Wallis test followed by a post hoc comparison using the Mann‒Whitney U test. A *P* value < 0.005 was considered significant with a Bonferroni correction. Because non-normally distributed data, including MFI scores and PPSV, were constants in the Sham and Dex-sham groups, these data were presented as median (interquartile range) and compared among the Control-IR, Dex-IR, and IR-Dex groups using the Kruskal‒Wallis test followed by a post hoc comparison using the Mann‒Whitney U test. A *P* value < 0.017 was considered significant with a Bonferroni correction.

## Data Availability

The datasets generated during and/or analysed during the current study are available from the corresponding author on reasonable request.
